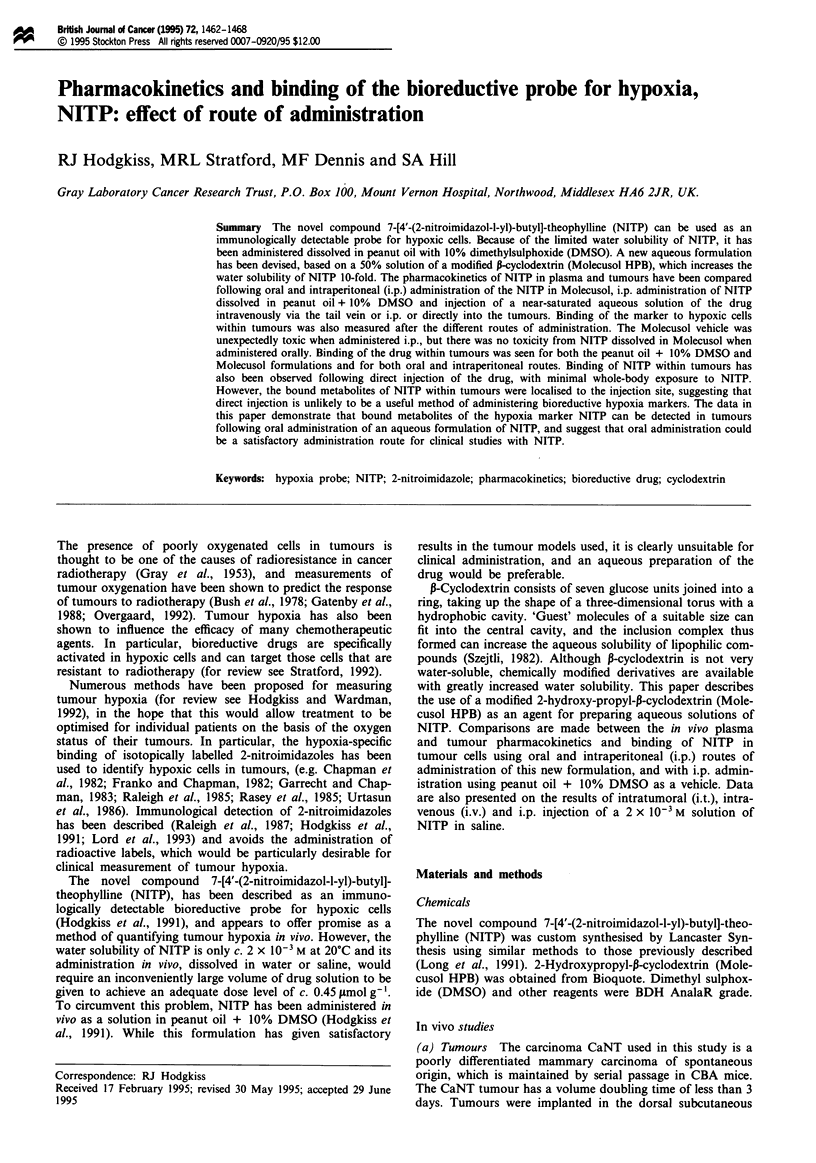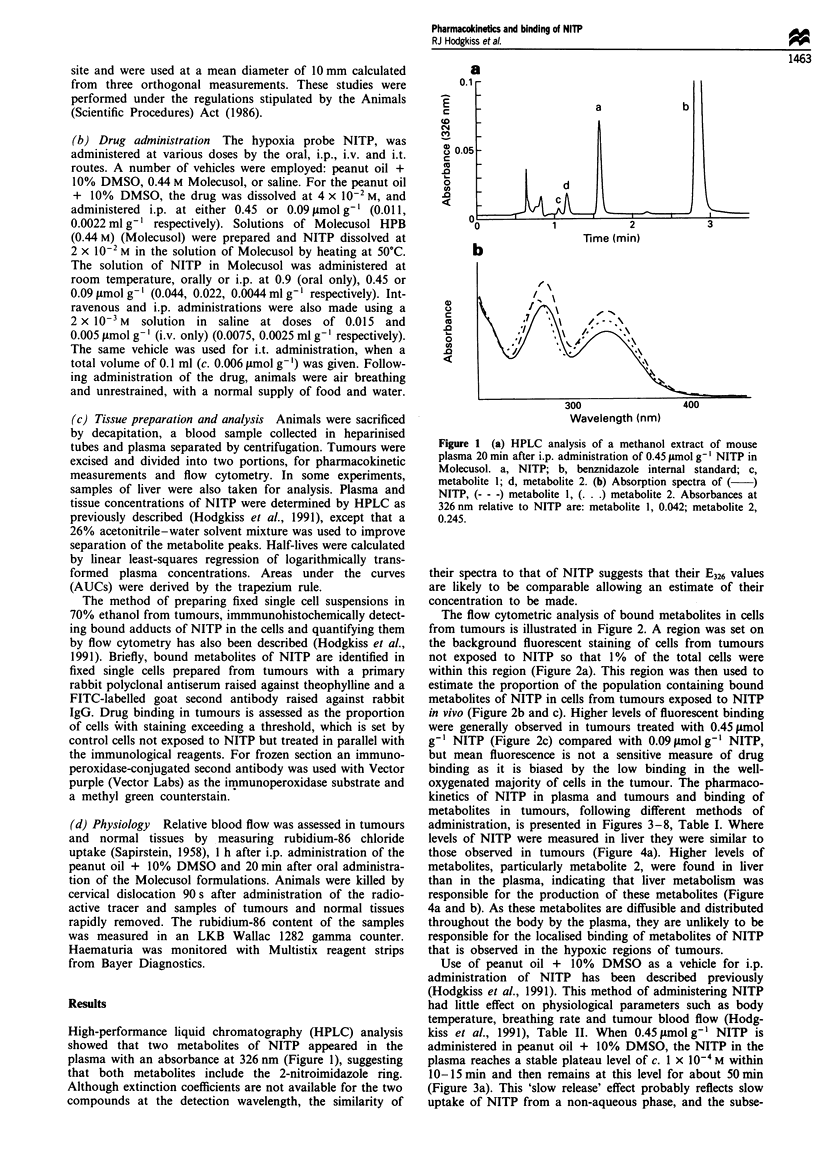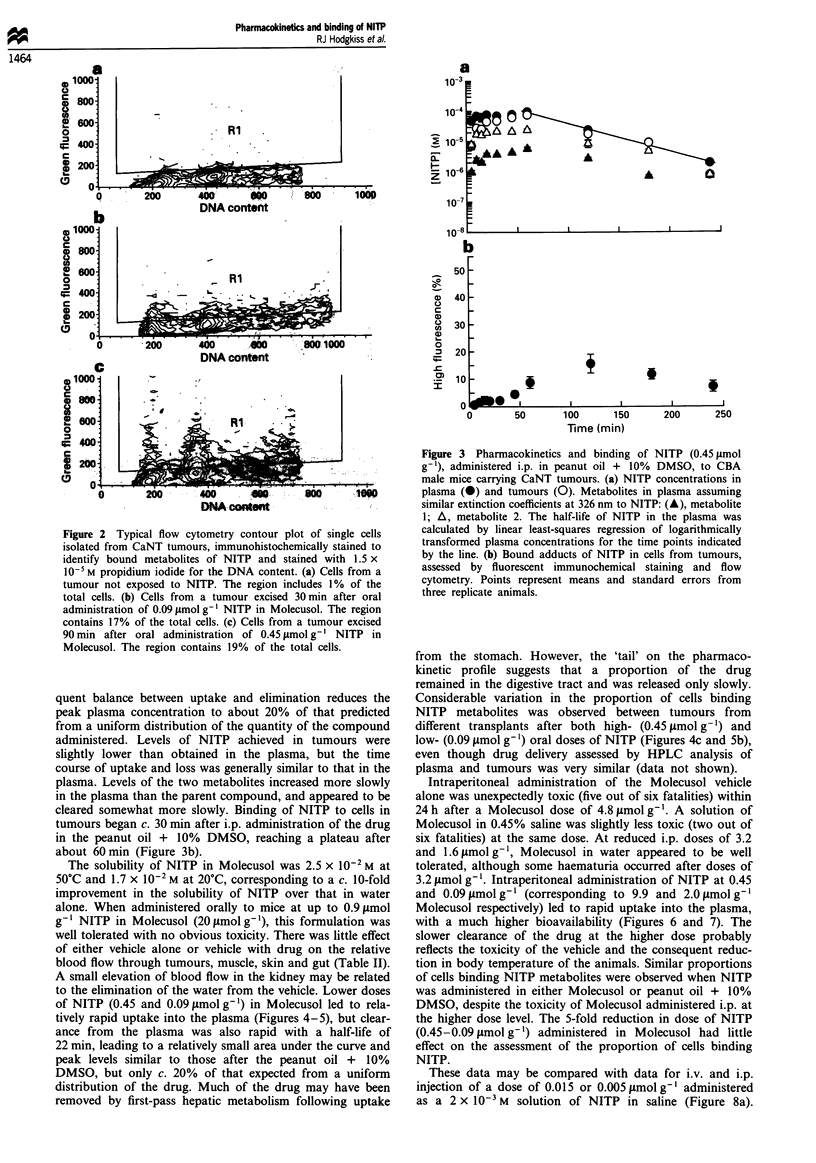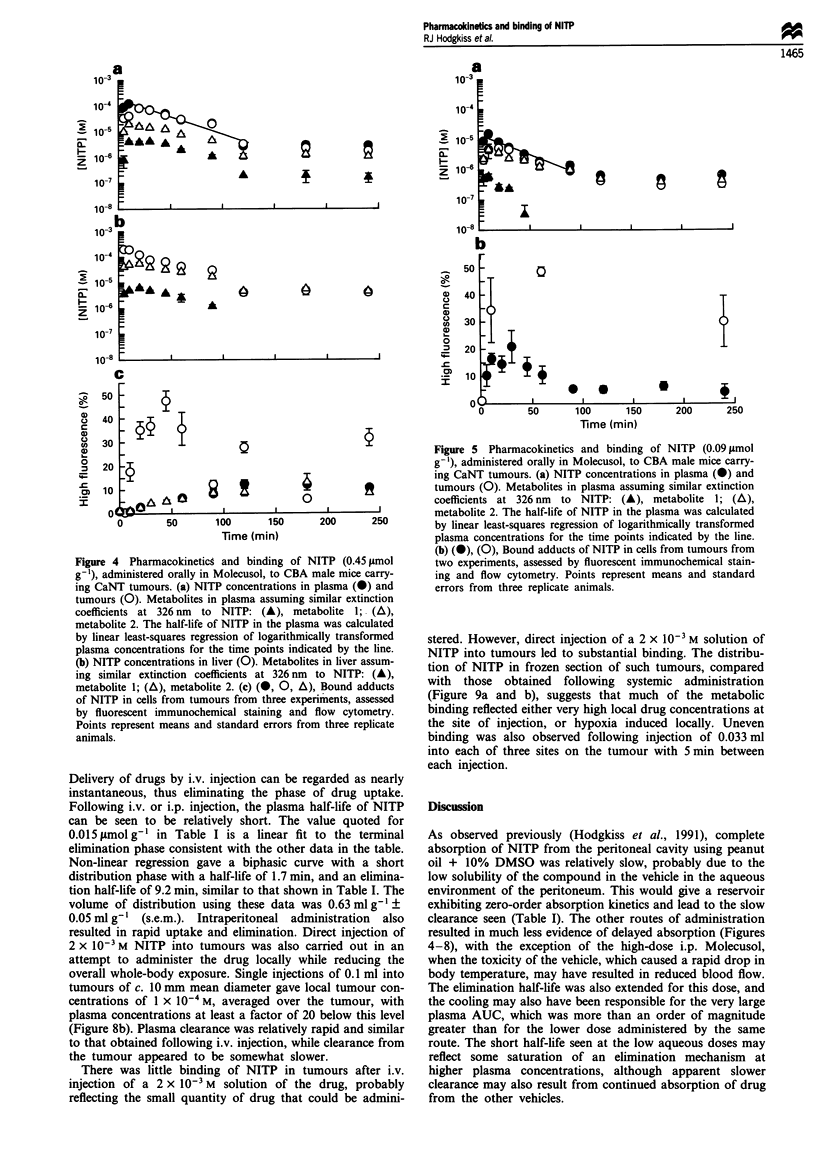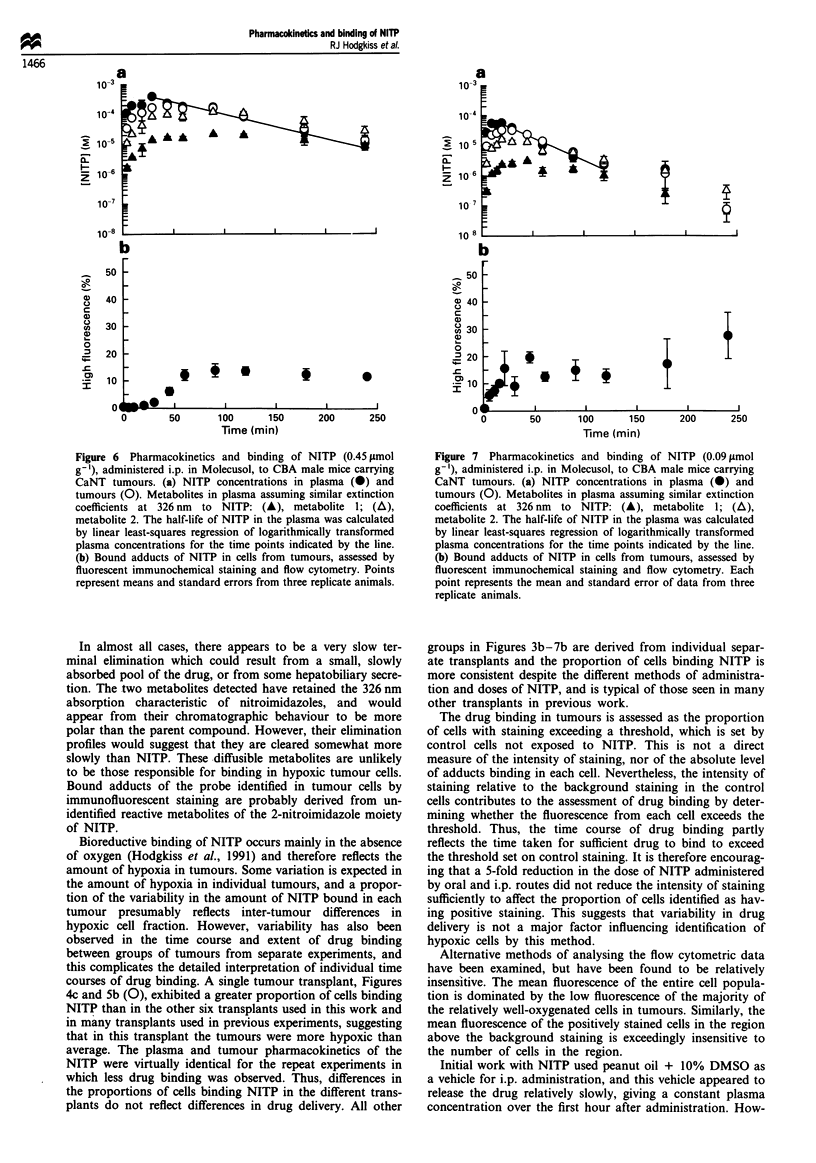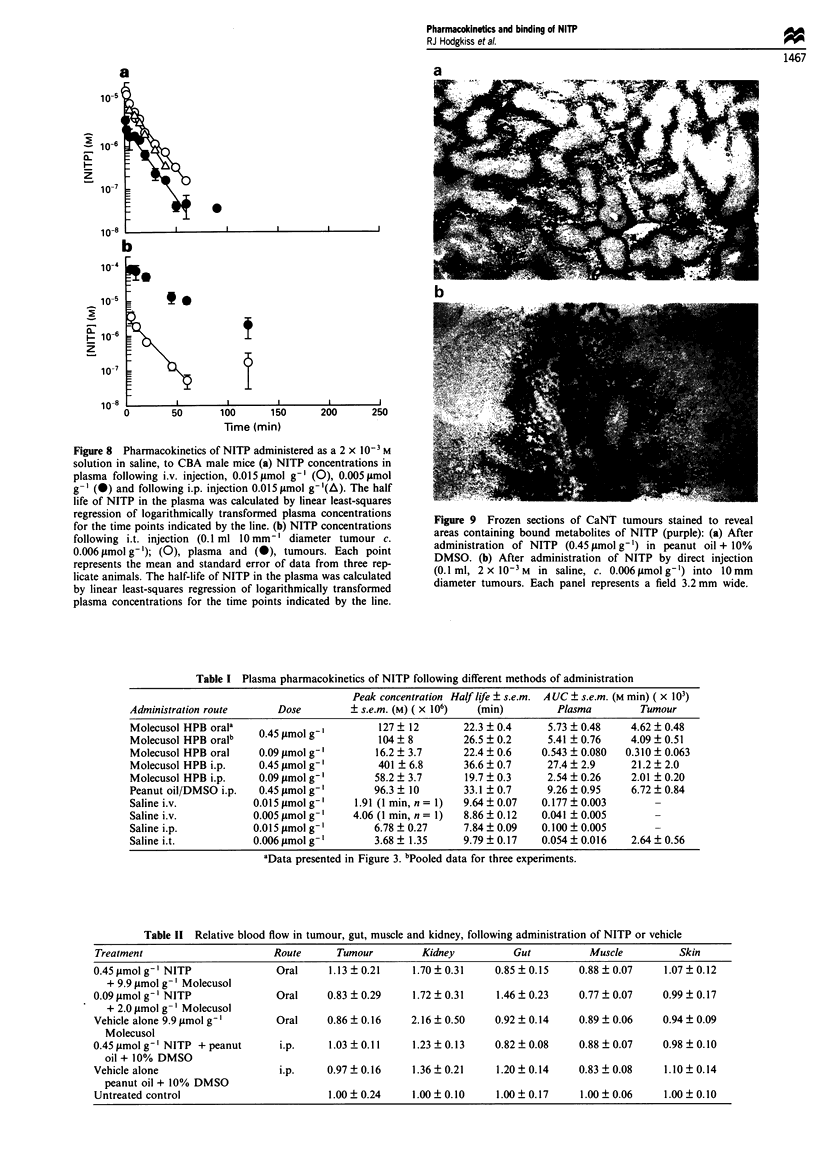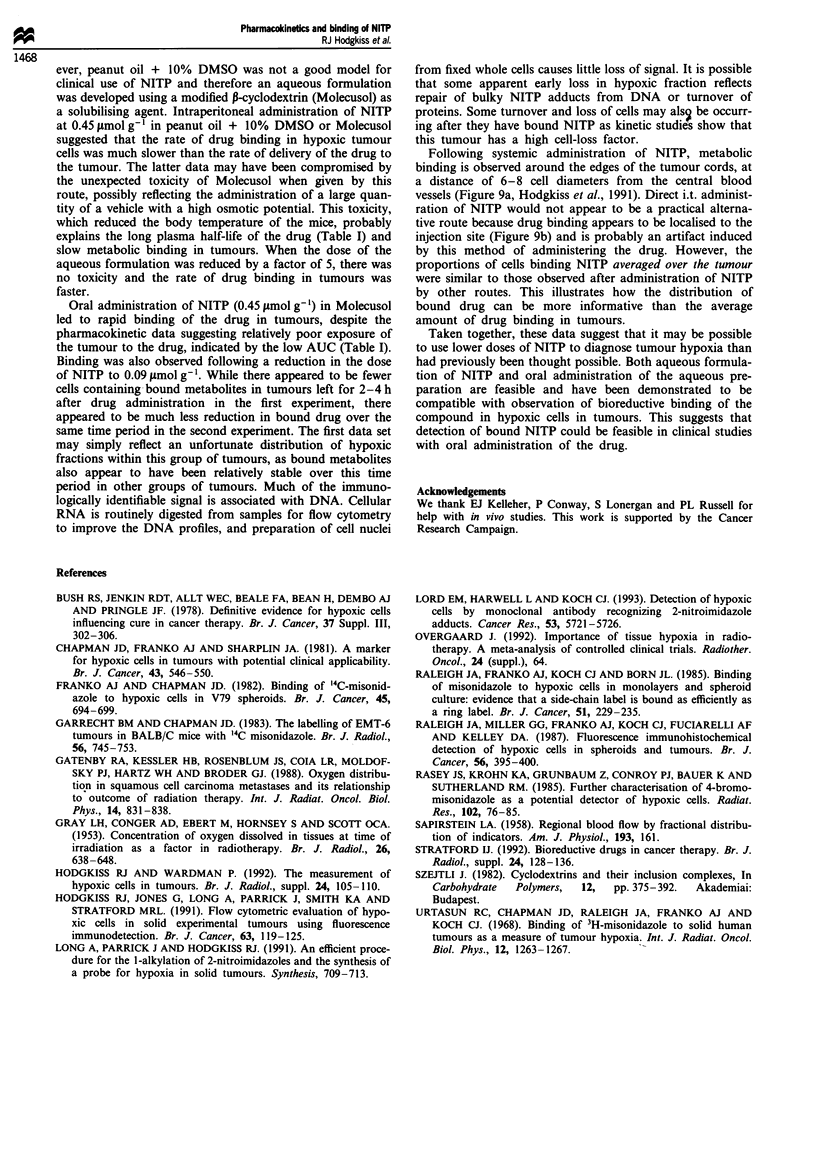# Pharmacokinetics and binding of the bioreductive probe for hypoxia, NITP: effect of route of administration.

**DOI:** 10.1038/bjc.1995.530

**Published:** 1995-12

**Authors:** R. J. Hodgkiss, M. R. Stratford, M. F. Dennis, S. A. Hill

**Affiliations:** Gray Laboratory Cancer Research Trust, Mount Vernon Hospital, Northwood, Middlesex, UK.

## Abstract

**Images:**


British Journal of Cancer (1995) 72, 1462-1468
© 1995 Stockton Press All rights reserved 0007-0920/95 $12.00

### Pharmacokinetics and binding of the bioreductive probe for hypoxia, NITP: effect of route of administration

RJ Hodgkiss, MRL Stratford, MF Dennis and SA Hill

*Gray Laboratory Cancer Research Trust, P.O. Box 100, Mount Vernon Hospital, Northwood, Middlesex HA6 2JR, UK.*

**Summary** The novel compound 7-[4'-(2-nitroimidazol-l-yl)-butyl]-theophylline (NITP) can be used as an immunologically detectable probe for hypoxic cells. Because of the limited water solubility of NITP, it has been administered dissolved in peanut oil with 10% dimethylsulphoxide (DMSO). A new aqueous formulation has been devised, based on a 50% solution of a modified β-cyclodextrin (Molecusol HPB), which increases the water solubility of NITP 10-fold. The pharmacokinetics of NITP in plasma and tumours have been compared following oral and intraperitoneal (i.p.) administration of the NITP in Molecusol, i.p. administration of NITP dissolved in peanut oil + 10% DMSO and injection of a near-saturated aqueous solution of the drug intravenously via the tail vein or i.p. or directly into the tumours. Binding of the marker to hypoxic cells within tumours was also measured after the different routes of administration. The Molecusol vehicle was unexpectedly toxic when administered i.p., but there was no toxicity from NITP dissolved in Molecusol when administered orally. Binding of the drug within tumours was seen for both the peanut oil + 10% DMSO and Molecusol formulations and for both oral and intraperitoneal routes. Binding of NITP within tumours has also been observed following direct injection of the drug, with minimal whole-body exposure to NITP. However, the bound metabolites of NITP within tumours were localised to the injection site, suggesting that direct injection is unlikely to be a useful method of administering bioreductive hypoxia markers. The data in this paper demonstrate that bound metabolites of the hypoxia marker NITP can be detected in tumours following oral administration of an aqueous formulation of NITP, and suggest that oral administration could be a satisfactory administration route for clinical studies with NITP.

**Keywords:** hypoxia probe; NITP; 2-nitroimidazole; pharmacokinetics; bioreductive drug; cyclodextrin

The presence of poorly oxygenated cells in tumours is thought to be one of the causes of radioresistance in cancer radiotherapy (Gray *et al.*, 1953), and measurements of tumour oxygenation have been shown to predict the response of tumours to radiotherapy (Bush *et al.*, 1978; Gatenby *et al.*, 1988; Overgaard, 1992). Tumour hypoxia has also been shown to influence the efficacy of many chemotherapeutic agents. In particular, bioreductive drugs are specifically activated in hypoxic cells and can target those cells that are resistant to radiotherapy (for review see Stratford, 1992).

Numerous methods have been proposed for measuring tumour hypoxia (for review see Hodgkiss and Wardman, 1992), in the hope that this would allow treatment to be optimised for individual patients on the basis of the oxygen status of their tumours. In particular, the hypoxia-specific binding of isotopically labelled 2-nitroimidazoles has been used to identify hypoxic cells in tumours, (e.g. Chapman *et al.*, 1982; Franko and Chapman, 1982; Garrecht and Chapman, 1983; Raleigh *et al.*, 1985; Rasey *et al.*, 1985; Urtasun *et al.*, 1986). Immunological detection of 2-nitroimidazoles has been described (Raleigh *et al.*, 1987; Hodgkiss *et al.*, 1991; Lord *et al.*, 1993) and avoids the administration of radioactive labels, which would be particularly desirable for clinical measurement of tumour hypoxia.

The novel compound 7-[4'-(2-nitroimidazol-l-yl)-butyl]-theophylline (NITP), has been described as an immunologically detectable bioreductive probe for hypoxic cells (Hodgkiss *et al.*, 1991), and appears to offer promise as a method of quantifying tumour hypoxia *in vivo*. However, the water solubility of NITP is only *c.* $2 \times 10^{-3}$ M at 20°C and its administration *in vivo*, dissolved in water or saline, would require an inconveniently large volume of drug solution to be given to achieve an adequate dose level of *c.* 0.45 $\mu$mol $g^{-1}$. To circumvent this problem, NITP has been administered *in vivo* as a solution in peanut oil + 10% DMSO (Hodgkiss *et al.*, 1991). While this formulation has given satisfactory results in the tumour models used, it is clearly unsuitable for clinical administration, and an aqueous preparation of the drug would be preferable.

β-Cyclodextrin consists of seven glucose units joined into a ring, taking up the shape of a three-dimensional torus with a hydrophobic cavity. 'Guest' molecules of a suitable size can fit into the central cavity, and the inclusion complex thus formed can increase the aqueous solubility of lipophilic compounds (Szejtli, 1982). Although β-cyclodextrin is not very water-soluble, chemically modified derivatives are available with greatly increased water solubility. This paper describes the use of a modified 2-hydroxy-propyl-β-cyclodextrin (Molecusol HPB) as an agent for preparing aqueous solutions of NITP. Comparisons are made between the *in vivo* plasma and tumour pharmacokinetics and binding of NITP in tumour cells using oral and intraperitoneal (i.p.) routes of administration of this new formulation, and with i.p. administration using peanut oil + 10% DMSO as a vehicle. Data are also presented on the results of intratumoral (i.t.), intravenous (i.v.) and i.p. injection of a $2 \times 10^{-3}$ M solution of NITP in saline.

### Materials and methods

### *Chemicals*

The novel compound 7-[4'-(2-nitroimidazol-l-yl)-butyl]-theophylline (NITP) was custom synthesised by Lancaster Synthesis using similar methods to those previously described (Long *et al.*, 1991). 2-Hydroxypropyl-β-cyclodextrin (Molecusol HPB) was obtained from Bioquote. Dimethyl sulphoxide (DMSO) and other reagents were BDH AnalaR grade.

### In vivo *studies*

*(a) Tumours* The carcinoma CaNT used in this study is a poorly differentiated mammary carcinoma of spontaneous origin, which is maintained by serial passage in CBA mice. The CaNT tumour has a volume doubling time of less than 3 days. Tumours were implanted in the dorsal subcutaneous

Correspondence: RJ Hodgkiss
Received 17 February 1995; revised 30 May 1995; accepted 29 June 1995

site and were used at a mean diameter of 10 mm calculated from three orthogonal measurements. These studies were performed under the regulations stipulated by the Animals (Scientific Procedures) Act (1986).

*(b) Drug administration* The hypoxia probe NITP, was administered at various doses by the oral, i.p., i.v. and i.t. routes. A number of vehicles were employed: peanut oil + 10% DMSO, 0.44 M Molecusol, or saline. For the peanut oil + 10% DMSO, the drug was dissolved at $4 \times 10^{-2}$ M, and administered i.p. at either 0.45 or 0.09 μmol $g^{-1}$ (0.011, 0.0022 ml $g^{-1}$ respectively). Solutions of Molecusol HPB (0.44 M) (Molecusol) were prepared and NITP dissolved at $2 \times 10^{-2}$ M in the solution of Molecusol by heating at 50°C. The solution of NITP in Molecusol was administered at room temperature, orally or i.p. at 0.9 (oral only), 0.45 or 0.09 μmol $g^{-1}$ (0.044, 0.022, 0.0044 ml $g^{-1}$ respectively). Intravenous and i.p. administrations were also made using a $2 \times 10^{-3}$ M solution in saline at doses of 0.015 and 0.005 μmol $g^{-1}$ (i.v. only) (0.0075, 0.0025 ml $g^{-1}$ respectively). The same vehicle was used for i.t. administration, when a total volume of 0.1 ml (*c.* 0.006 μmol $g^{-1}$) was given. Following administration of the drug, animals were air breathing and unrestrained, with a normal supply of food and water.

*(c) Tissue preparation and analysis* Animals were sacrificed by decapitation, a blood sample collected in heparinised tubes and plasma separated by centrifugation. Tumours were excised and divided into two portions, for pharmacokinetic measurements and flow cytometry. In some experiments, samples of liver were also taken for analysis. Plasma and tissue concentrations of NITP were determined by HPLC as previously described (Hodgkiss *et al.*, 1991), except that a 26% acetonitrile–water solvent mixture was used to improve separation of the metabolite peaks. Half-lives were calculated by linear least-squares regression of logarithmically transformed plasma concentrations. Areas under the curves (AUCs) were derived by the trapezium rule.

The method of preparing fixed single cell suspensions in 70% ethanol from tumours, immmunohistochemically detecting bound adducts of NITP in the cells and quantifying them by flow cytometry has also been described (Hodgkiss *et al.*, 1991). Briefly, bound metabolites of NITP are identified in fixed single cells prepared from tumours with a primary rabbit polyclonal antiserum raised against theophylline and a FITC-labelled goat second antibody raised against rabbit IgG. Drug binding in tumours is assessed as the proportion of cells with staining exceeding a threshold, which is set by control cells not exposed to NITP but treated in parallel with the immunological reagents. For frozen section an immunoperoxidase-conjugated second antibody was used with Vector purple (Vector Labs) as the immunoperoxidase substrate and a methyl green counterstain.

*(d) Physiology* Relative blood flow was assessed in tumours and normal tissues by measuring rubidium-86 chloride uptake (Sapirstein, 1958), 1 h after i.p. administration of the peanut oil + 10% DMSO and 20 min after oral administration of the Molecusol formulations. Animals were killed by cervical dislocation 90 s after administration of the radioactive tracer and samples of tumours and normal tissues rapidly removed. The rubidium-86 content of the samples was measured in an LKB Wallac 1282 gamma counter. Haematuria was monitored with Multistix reagent strips from Bayer Diagnostics.

### Results

High-performance liquid chromatography (HPLC) analysis showed that two metabolites of NITP appeared in the plasma with an absorbance at 326 nm (Figure 1), suggesting that both metabolites include the 2-nitroimidazole ring. Although extinction coefficients are not available for the two compounds at the detection wavelength, the similarity of their spectra to that of NITP suggests that their $E_{326}$ values are likely to be comparable allowing an estimate of their concentration to be made.

**Figure 1** (**a**) HPLC analysis of a methanol extract of mouse plasma 20 min after i.p. administration of 0.45 μmol $g^{-1}$ NITP in Molecusol. a, NITP; b, benznidazole internal standard; c, metabolite 1; d, metabolite 2. (**b**) Absorption spectra of (——) NITP, (- - -) metabolite 1, (. . .) metabolite 2. Absorbances at 326 nm relative to NITP are: metabolite 1, 0.042; metabolite 2, 0.245.

The flow cytometric analysis of bound metabolites in cells from tumours is illustrated in Figure 2. A region was set on the background fluorescent staining of cells from tumours not exposed to NITP so that 1% of the total cells were within this region (Figure 2a). This region was then used to estimate the proportion of the population containing bound metabolites of NITP in cells from tumours exposed to NITP *in vivo* (Figure 2b and c). Higher levels of fluorescent binding were generally observed in tumours treated with 0.45 μmol $g^{-1}$ NITP (Figure 2c) compared with 0.09 μmol $g^{-1}$ NITP, but mean fluorescence is not a sensitive measure of drug binding as it is biased by the low binding in the well-oxygenated majority of cells in the tumour. The pharmacokinetics of NITP in plasma and tumours and binding of metabolites in tumours, following different methods of administration, is presented in Figures 3–8, Table I. Where levels of NITP were measured in liver they were similar to those observed in tumours (Figure 4a). Higher levels of metabolites, particularly metabolite 2, were found in liver than in the plasma, indicating that liver metabolism was responsible for the production of these metabolites (Figure 4a and b). As these metabolites are diffusible and distributed throughout the body by the plasma, they are unlikely to be responsible for the localised binding of metabolites of NITP that is observed in the hypoxic regions of tumours.

Use of peanut oil + 10% DMSO as a vehicle for i.p. administration of NITP has been described previously (Hodgkiss *et al.*, 1991). This method of administering NITP had little effect on physiological parameters such as body temperature, breathing rate and tumour blood flow (Hodgkiss *et al.*, 1991), Table II. When 0.45 μmol $g^{-1}$ NITP is administered in peanut oil + 10% DMSO, the NITP in the plasma reaches a stable plateau level of *c.* $1 \times 10^{-4}$ M within 10–15 min and then remains at this level for about 50 min (Figure 3a). This 'slow release' effect probably reflects slow uptake of NITP from a non-aqueous phase, and the subse-

**Figure 2** Typical flow cytometry contour plot of single cells isolated from CaNT tumours, immunohistochemically stained to identify bound metabolites of NITP and stained with $1.5 \times 10^{-5}$ M propidium iodide for the DNA content. (**a**) Cells from a tumour not exposed to NITP. The region includes 1% of the total cells. (**b**) Cells from a tumour excised 30 min after oral administration of 0.09 $\mu$mol g$^{-1}$ NITP in Molecusol. The region contains 17% of the total cells. (**c**) Cells from a tumour excised 90 min after oral administration of 0.45 $\mu$mol g$^{-1}$ NITP in Molecusol. The region contains 19% of the total cells.

**Figure 3** Pharmacokinetics and binding of NITP (0.45 $\mu$mol g$^{-1}$), administered i.p. in peanut oil + 10% DMSO, to CBA male mice carrying CaNT tumours. (**a**) NITP concentrations in plasma (●) and tumours (○). Metabolites in plasma assuming similar extinction coefficients at 326 nm to NITP: (▲), metabolite 1; △, metabolite 2. The half-life of NITP in the plasma was calculated by linear least-squares regression of logarithmically transformed plasma concentrations for the time points indicated by the line. (**b**) Bound adducts of NITP in cells from tumours, assessed by fluorescent immunochemical staining and flow cytometry. Points represent means and standard errors from three replicate animals.

quent balance between uptake and elimination reduces the peak plasma concentration to about 20% of that predicted from a uniform distribution of the quantity of the compound administered. Levels of NITP achieved in tumours were slightly lower than obtained in the plasma, but the time course of uptake and loss was generally similar to that in the plasma. Levels of the two metabolites increased more slowly in the plasma than the parent compound, and appeared to be cleared somewhat more slowly. Binding of NITP to cells in tumours began *c*. 30 min after i.p. administration of the drug in the peanut oil + 10% DMSO, reaching a plateau after about 60 min (Figure 3b).

The solubility of NITP in Molecusol was $2.5 \times 10^{-2}$ M at 50°C and $1.7 \times 10^{-2}$ M at 20°C, corresponding to a *c*. 10-fold improvement in the solubility of NITP over that in water alone. When administered orally to mice at up to 0.9 $\mu$mol g$^{-1}$ NITP in Molecusol (20 $\mu$mol g$^{-1}$), this formulation was well tolerated with no obvious toxicity. There was little effect of either vehicle alone or vehicle with drug on the relative blood flow through tumours, muscle, skin and gut (Table II). A small elevation of blood flow in the kidney may be related to the elimination of the water from the vehicle. Lower doses of NITP (0.45 and 0.09 $\mu$mol g$^{-1}$) in Molecusol led to relatively rapid uptake into the plasma (Figures 4–5), but clearance from the plasma was also rapid with a half-life of 22 min, leading to a relatively small area under the curve and peak levels similar to those after the peanut oil + 10% DMSO, but only *c*. 20% of that expected from a uniform distribution of the drug. Much of the drug may have been removed by first-pass hepatic metabolism following uptake from the stomach. However, the 'tail' on the pharmacokinetic profile suggests that a proportion of the drug remained in the digestive tract and was released only slowly. Considerable variation in the proportion of cells binding NITP metabolites was observed between tumours from different transplants after both high- (0.45 $\mu$mol g$^{-1}$) and low- (0.09 $\mu$mol g$^{-1}$) oral doses of NITP (Figures 4c and 5b), even though drug delivery assessed by HPLC analysis of plasma and tumours was very similar (data not shown).

Intraperitoneal administration of the Molecusol vehicle alone was unexpectedly toxic (five out of six fatalities) within 24 h after a Molecusol dose of 4.8 $\mu$mol g$^{-1}$. A solution of Molecusol in 0.45% saline was slightly less toxic (two out of six fatalities) at the same dose. At reduced i.p. doses of 3.2 and 1.6 $\mu$mol g$^{-1}$, Molecusol in water appeared to be well tolerated, although some haematuria occurred after doses of 3.2 $\mu$mol g$^{-1}$. Intraperitoneal administration of NITP at 0.45 and 0.09 $\mu$mol g$^{-1}$ (corresponding to 9.9 and 2.0 $\mu$mol g$^{-1}$ Molecusol respectively) led to rapid uptake into the plasma, with a much higher bioavailability (Figures 6 and 7). The slower clearance of the drug at the higher dose probably reflects the toxicity of the vehicle and the consequent reduction in body temperature of the animals. Similar proportions of cells binding NITP metabolites were observed when NITP was administered in either Molecusol or peanut oil + 10% DMSO, despite the toxicity of Molecusol administered i.p. at the higher dose level. The 5-fold reduction in dose of NITP (0.45–0.09 $\mu$mol g$^{-1}$) administered in Molecusol had little effect on the assessment of the proportion of cells binding NITP.

These data may be compared with data for i.v. and i.p. injection of a dose of 0.015 or 0.005 $\mu$mol g$^{-1}$ administered as a $2 \times 10^{-3}$ M solution of NITP in saline (Figure 8a).

**Figure 4** Pharmacokinetics and binding of NITP (0.45 μmol $g^{-1}$), administered orally in Molecusol, to CBA male mice carrying CaNT tumours. (**a**) NITP concentrations in plasma (●) and tumours (○). Metabolites in plasma assuming similar extinction coefficients at 326 nm to NITP: (▲), metabolite 1; (△), metabolite 2. The half-life of NITP in the plasma was calculated by linear least-squares regression of logarithmically transformed plasma concentrations for the time points indicated by the line. (**b**) NITP concentrations in liver (○). Metabolites in liver assuming similar extinction coefficients at 326 nm to NITP: (▲), metabolite 1; (△), metabolite 2. (**c**) (●, ○, △), Bound adducts of NITP in cells from tumours from three experiments, assessed by fluorescent immunochemical staining and flow cytometry. Points represent means and standard errors from three replicate animals.

**Figure 5** Pharmacokinetics and binding of NITP (0.09 μmol $g^{-1}$), administered orally in Molecusol, to CBA male mice carrying CaNT tumours. (**a**) NITP concentrations in plasma (●) and tumours (○). Metabolites in plasma assuming similar extinction coefficients at 326 nm to NITP: (▲), metabolite 1; (△), metabolite 2. The half-life of NITP in the plasma was calculated by linear least-squares regression of logarithmically transformed plasma concentrations for the time points indicated by the line. (**b**) (●), (○), Bound adducts of NITP in cells from tumours from two experiments, assessed by fluorescent immunochemical staining and flow cytometry. Points represent means and standard errors from three replicate animals.

Delivery of drugs by i.v. injection can be regarded as nearly instantaneous, thus eliminating the phase of drug uptake. Following i.v. or i.p. injection, the plasma half-life of NITP can be seen to be relatively short. The value quoted for 0.015 μmol $g^{-1}$ in Table I is a linear fit to the terminal elimination phase consistent with the other data in the table. Non-linear regression gave a biphasic curve with a short distribution phase with a half-life of 1.7 min, and an elimination half-life of 9.2 min, similar to that shown in Table I. The volume of distribution using these data was 0.63 ml $g^{-1}$ ± 0.05 ml $g^{-1}$ (s.e.m.). Intraperitoneal administration also resulted in rapid uptake and elimination. Direct injection of $2 \times 10^{-3}$ M NITP into tumours was also carried out in an attempt to administer the drug locally while reducing the overall whole-body exposure. Single injections of 0.1 ml into tumours of *c*. 10 mm mean diameter gave local tumour concentrations of $1 \times 10^{-4}$ M, averaged over the tumour, with plasma concentrations at least a factor of 20 below this level (Figure 8b). Plasma clearance was relatively rapid and similar to that obtained following i.v. injection, while clearance from the tumour appeared to be somewhat slower.

There was little binding of NITP in tumours after i.v. injection of a $2 \times 10^{-3}$ M solution of the drug, probably reflecting the small quantity of drug that could be administered. However, direct injection of a $2 \times 10^{-3}$ M solution of NITP into tumours led to substantial binding. The distribution of NITP in frozen section of such tumours, compared with those obtained following systemic administration (Figure 9a and b), suggests that much of the metabolic binding reflected either very high local drug concentrations at the site of injection, or hypoxia induced locally. Uneven binding was also observed following injection of 0.033 ml into each of three sites on the tumour with 5 min between each injection.

### Discussion

As observed previously (Hodgkiss *et al.*, 1991), complete absorption of NITP from the peritoneal cavity using peanut oil + 10% DMSO was relatively slow, probably due to the low solubility of the compound in the vehicle in the aqueous environment of the peritoneum. This would give a reservoir exhibiting zero-order absorption kinetics and lead to the slow clearance seen (Table I). The other routes of administration resulted in much less evidence of delayed absorption (Figures 4–8), with the exception of the high-dose i.p. Molecusol, when the toxicity of the vehicle, which caused a rapid drop in body temperature, may have resulted in reduced blood flow. The elimination half-life was also extended for this dose, and the cooling may also have been responsible for the very large plasma AUC, which was more than an order of magnitude greater than for the lower dose administered by the same route. The short half-life seen at the low aqueous doses may reflect some saturation of an elimination mechanism at higher plasma concentrations, although apparent slower clearance may also result from continued absorption of drug from the other vehicles.

**Figure 6** Pharmacokinetics and binding of NITP (0.45 μmol $g^{-1}$), administered i.p. in Molecusol, to CBA male mice carrying CaNT tumours. (**a**) NITP concentrations in plasma (●) and tumours (○). Metabolites in plasma assuming similar extinction coefficients at 326 nm to NITP: (▲), metabolite 1; (△), metabolite 2. The half-life of NITP in the plasma was calculated by linear least-squares regression of logarithmically transformed plasma concentrations for the time points indicated by the line. (**b**) Bound adducts of NITP in cells from tumours, assessed by fluorescent immunochemical staining and flow cytometry. Points represent means and standard errors from three replicate animals.

**Figure 7** Pharmacokinetics and binding of NITP (0.09 μmol $g^{-1}$), administered i.p. in Molecusol, to CBA male mice carrying CaNT tumours. (**a**) NITP concentrations in plasma (●) and tumours (○). Metabolites in plasma assuming similar extinction coefficients at 326 nm to NITP: (▲), metabolite 1; (△), metabolite 2. The half-life of NITP in the plasma was calculated by linear least-squares regression of logarithmically transformed plasma concentrations for the time points indicated by the line. (**b**) Bound adducts of NITP in cells from tumours, assessed by fluorescent immunochemical staining and flow cytometry. Each point represents the mean and standard error of data from three replicate animals.

In almost all cases, there appears to be a very slow terminal elimination which could result from a small, slowly absorbed pool of the drug, or from some hepatobiliary secretion. The two metabolites detected have retained the 326 nm absorption characteristic of nitroimidazoles, and would appear from their chromatographic behaviour to be more polar than the parent compound. However, their elimination profiles would suggest that they are cleared somewhat more slowly than NITP. These diffusible metabolites are unlikely to be those responsible for binding in hypoxic tumour cells. Bound adducts of the probe identified in tumour cells by immunofluorescent staining are probably derived from unidentified reactive metabolites of the 2-nitroimidazole moiety of NITP.

Bioreductive binding of NITP occurs mainly in the absence of oxygen (Hodgkiss *et al.*, 1991) and therefore reflects the amount of hypoxia in tumours. Some variation is expected in the amount of hypoxia in individual tumours, and a proportion of the variability in the amount of NITP bound in each tumour presumably reflects inter-tumour differences in hypoxic cell fraction. However, variability has also been observed in the time course and extent of drug binding between groups of tumours from separate experiments, and this complicates the detailed interpretation of individual time courses of drug binding. A single tumour transplant, Figures 4c and 5b (○), exhibited a greater proportion of cells binding NITP than in the other six transplants used in this work and in many transplants used in previous experiments, suggesting that in this transplant the tumours were more hypoxic than average. The plasma and tumour pharmacokinetics of the NITP were virtually identical for the repeat experiments in which less drug binding was observed. Thus, differences in the proportions of cells binding NITP in the different transplants do not reflect differences in drug delivery. All other groups in Figures 3b–7b are derived from individual separate transplants and the proportion of cells binding NITP is more consistent despite the different methods of administration and doses of NITP, and is typical of those seen in many other transplants in previous work.

The drug binding in tumours is assessed as the proportion of cells with staining exceeding a threshold, which is set by control cells not exposed to NITP. This is not a direct measure of the intensity of staining, nor of the absolute level of adducts binding in each cell. Nevertheless, the intensity of staining relative to the background staining in the control cells contributes to the assessment of drug binding by determining whether the fluorescence from each cell exceeds the threshold. Thus, the time course of drug binding partly reflects the time taken for sufficient drug to bind to exceed the threshold set on control staining. It is therefore encouraging that a 5-fold reduction in the dose of NITP administered by oral and i.p. routes did not reduce the intensity of staining sufficiently to affect the proportion of cells identified as having positive staining. This suggests that variability in drug delivery is not a major factor influencing identification of hypoxic cells by this method.

Alternative methods of analysing the flow cytometric data have been examined, but have been found to be relatively insensitive. The mean fluorescence of the entire cell population is dominated by the low fluorescence of the majority of the relatively well-oxygenated cells in tumours. Similarly, the mean fluorescence of the positively stained cells in the region above the background staining is exceedingly insensitive to the number of cells in the region.

Initial work with NITP used peanut oil + 10% DMSO as a vehicle for i.p. administration, and this vehicle appeared to release the drug relatively slowly, giving a constant plasma concentration over the first hour after administration. How-

**Figure 8** Pharmacokinetics of NITP administered as a $2 \times 10^{-3}$ M solution in saline, to CBA male mice (**a**) NITP concentrations in plasma following i.v. injection, 0.015 µmol $g^{-1}$ (○), 0.005 µmol $g^{-1}$ (●) and following i.p. injection 0.015 µmol $g^{-1}$(Δ). The half life of NITP in the plasma was calculated by linear least-squares regression of logarithmically transformed plasma concentrations for the time points indicated by the line. (**b**) NITP concentrations following i.t. injection (0.1 ml 10 $mm^{-1}$ diameter tumour *c.* 0.006 µmol $g^{-1}$); (○), plasma and (●), tumours. Each point represents the mean and standard error of data from three replicate animals. The half-life of NITP in the plasma was calculated by linear least-squares regression of logarithmically transformed plasma concentrations for the time points indicated by the line.

**Figure 9** Frozen sections of CaNT tumours stained to reveal areas containing bound metabolites of NITP (purple): (**a**) After administration of NITP (0.45 µmol $g^{-1}$) in peanut oil + 10% DMSO. (**b**) After administration of NITP by direct injection (0.1 ml, $2 \times 10^{-3}$ M in saline, *c.* 0.006 µmol $g^{-1}$) into 10 mm diameter tumours. Each panel represents a field 3.2 mm wide.

**Table I** Plasma pharmacokinetics of NITP following different methods of administration

| Administration route | Dose | *Peak concentration ± s.e.m.* (M) ( × $10^6$) | *Half life ± s.e.m.* (min) | *AUC ± s.e.m.* (M min) ( × $10^3$) *Plasma* | *Tumour* |
|---|---|---|---|---|---|
| Molecusol HPB oral[a] | 0.45 µmol $g^{-1}$ | 127 ± 12 | 22.3 ± 0.4 | 5.73 ± 0.48 | 4.62 ± 0.48 |
| Molecusol HPB oral[b] | | 104 ± 8 | 26.5 ± 0.2 | 5.41 ± 0.76 | 4.09 ± 0.51 |
| Molecusol HPB oral | 0.09 µmol $g^{-1}$ | 16.2 ± 3.7 | 22.4 ± 0.6 | 0.543 ± 0.080 | 0.310 ± 0.063 |
| Molecusol HPB i.p. | 0.45 µmol $g^{-1}$ | 401 ± 6.8 | 36.6 ± 0.7 | 27.4 ± 2.9 | 21.2 ± 2.0 |
| Molecusol HPB i.p. | 0.09 µmol $g^{-1}$ | 58.2 ± 3.7 | 19.7 ± 0.3 | 2.54 ± 0.26 | 2.01 ± 0.20 |
| Peanut oil/DMSO i.p. | 0.45 µmol $g^{-1}$ | 96.3 ± 10 | 33.1 ± 0.7 | 9.26 ± 0.95 | 6.72 ± 0.84 |
| Saline i.v. | 0.015 µmol $g^{-1}$ | 1.91 (1 min, $n = 1$) | 9.64 ± 0.07 | 0.177 ± 0.003 | – |
| Saline i.v. | 0.005 µmol $g^{-1}$ | 4.06 (1 min, $n = 1$) | 8.86 ± 0.12 | 0.041 ± 0.005 | – |
| Saline i.p. | 0.015 µmol $g^{-1}$ | 6.78 ± 0.27 | 7.84 ± 0.09 | 0.100 ± 0.005 | – |
| Saline i.t. | 0.006 µmol $g^{-1}$ | 3.68 ± 1.35 | 9.79 ± 0.17 | 0.054 ± 0.016 | 2.64 ± 0.56 |

[a]Data presented in Figure 3. [b]Pooled data for three experiments.

**Table II** Relative blood flow in tumour, gut, muscle and kidney, following administration of NITP or vehicle

| *Treatment* | *Route* | *Tumour* | *Kidney* | *Gut* | *Muscle* | *Skin* |
|---|---|---|---|---|---|---|
| 0.45 µmol $g^{-1}$ NITP + 9.9 µmol $g^{-1}$ Molecusol | Oral | 1.13 ± 0.21 | 1.70 ± 0.31 | 0.85 ± 0.15 | 0.88 ± 0.07 | 1.07 ± 0.12 |
| 0.09 µmol $g^{-1}$ NITP + 2.0 µmol $g^{-1}$ Molecusol | Oral | 0.83 ± 0.29 | 1.72 ± 0.31 | 1.46 ± 0.23 | 0.77 ± 0.07 | 0.99 ± 0.17 |
| Vehicle alone 9.9 µmol $g^{-1}$ Molecusol | Oral | 0.86 ± 0.16 | 2.16 ± 0.50 | 0.92 ± 0.14 | 0.89 ± 0.06 | 0.94 ± 0.09 |
| 0.45 µmol $g^{-1}$ NITP + peanut oil + 10% DMSO | i.p. | 1.03 ± 0.11 | 1.23 ± 0.13 | 0.82 ± 0.08 | 0.88 ± 0.07 | 0.98 ± 0.10 |
| Vehicle alone peanut oil + 10% DMSO | i.p. | 0.97 ± 0.16 | 1.36 ± 0.21 | 1.20 ± 0.14 | 0.83 ± 0.08 | 1.10 ± 0.14 |
| Untreated control | | 1.00 ± 0.24 | 1.00 ± 0.10 | 1.00 ± 0.17 | 1.00 ± 0.06 | 1.00 ± 0.10 |

ever, peanut oil + 10% DMSO was not a good model for clinical use of NITP and therefore an aqueous formulation was developed using a modified β-cyclodextrin (Molecusol) as a solubilising agent. Intraperitoneal administration of NITP at 0.45 $\mu mol\ g^{-1}$ in peanut oil + 10% DMSO or Molecusol suggested that the rate of drug binding in hypoxic tumour cells was much slower than the rate of delivery of the drug to the tumour. The latter data may have been compromised by the unexpected toxicity of Molecusol when given by this route, possibly reflecting the administration of a large quantity of a vehicle with a high osmotic potential. This toxicity, which reduced the body temperature of the mice, probably explains the long plasma half-life of the drug (Table I) and slow metabolic binding in tumours. When the dose of the aqueous formulation was reduced by a factor of 5, there was no toxicity and the rate of drug binding in tumours was faster.

Oral administration of NITP (0.45 $\mu mol\ g^{-1}$) in Molecusol led to rapid binding of the drug in tumours, despite the pharmacokinetic data suggesting relatively poor exposure of the tumour to the drug, indicated by the low AUC (Table I). Binding was also observed following a reduction in the dose of NITP to 0.09 $\mu mol\ g^{-1}$. While there appeared to be fewer cells containing bound metabolites in tumours left for 2–4 h after drug administration in the first experiment, there appeared to be much less reduction in bound drug over the same time period in the second experiment. The first data set may simply reflect an unfortunate distribution of hypoxic fractions within this group of tumours, as bound metabolites also appear to have been relatively stable over this time period in other groups of tumours. Much of the immunologically identifiable signal is associated with DNA. Cellular RNA is routinely digested from samples for flow cytometry to improve the DNA profiles, and preparation of cell nuclei from fixed whole cells causes little loss of signal. It is possible that some apparent early loss in hypoxic fraction reflects repair of bulky NITP adducts from DNA or turnover of proteins. Some turnover and loss of cells may also be occurring after they have bound NITP as kinetic studies show that this tumour has a high cell-loss factor.

Following systemic administration of NITP, metabolic binding is observed around the edges of the tumour cords, at a distance of 6–8 cell diameters from the central blood vessels (Figure 9a, Hodgkiss *et al.*, 1991). Direct i.t. administration of NITP would not appear to be a practical alternative route because drug binding appears to be localised to the injection site (Figure 9b) and is probably an artifact induced by this method of administering the drug. However, the proportions of cells binding NITP *averaged over the tumour* were similar to those observed after administration of NITP by other routes. This illustrates how the distribution of bound drug can be more informative than the average amount of drug binding in tumours.

Taken together, these data suggest that it may be possible to use lower doses of NITP to diagnose tumour hypoxia than had previously been thought possible. Both aqueous formulation of NITP and oral administration of the aqueous preparation are feasible and have been demonstrated to be compatible with observation of bioreductive binding of the compound in hypoxic cells in tumours. This suggests that detection of bound NITP could be feasible in clinical studies with oral administration of the drug.

**Acknowledgements**
We thank EJ Kelleher, P Conway, S Lonergan and PL Russell for help with *in vivo* studies. This work is supported by the Cancer Research Campaign.

### References

BUSH RS, JENKIN RDT, ALLT WEC, BEALE FA, BEAN H, DEMBO AJ AND PRINGLE JF. (1978). Definitive evidence for hypoxic cells influencing cure in cancer therapy. *Br. J. Cancer*, **37** Suppl. III, 302–306.

CHAPMAN JD, FRANKO AJ AND SHARPLIN JA. (1981). A marker for hypoxic cells in tumours with potential clinical applicability. *Br. J. Cancer*, **43**, 546–550.

FRANKO AJ AND CHAPMAN JD. (1982). Binding of $^{14}$C-misonidazole to hypoxic cells in V79 spheroids. *Br. J. Cancer*, **45**, 694–699.

GARRECHT BM AND CHAPMAN JD. (1983). The labelling of EMT-6 tumours in BALB/C mice with $^{14}$C misonidazole. *Br. J. Radiol.*, **56**, 745–753.

GATENBY RA, KESSLER HB, ROSENBLUM JS, COIA LR, MOLDOFSKY PJ, HARTZ WH AND BRODER GJ. (1988). Oxygen distribution in squamous cell carcinoma metastases and its relationship to outcome of radiation therapy. *Int. J. Radiat. Oncol. Biol. Phys.*, **14**, 831–838.

GRAY LH, CONGER AD, EBERT M, HORNSEY S AND SCOTT OCA. (1953). Concentration of oxygen dissolved in tissues at time of irradiation as a factor in radiotherapy. *Br. J. Radiol.*, **26**, 638–648.

HODGKISS RJ AND WARDMAN P. (1992). The measurement of hypoxic cells in tumours. *Br. J. Radiol.*, suppl. **24**, 105–110.

HODGKISS RJ, JONES G, LONG A, PARRICK J, SMITH KA AND STRATFORD MRL. (1991). Flow cytometric evaluation of hypoxic cells in solid experimental tumours using fluorescence immunodetection. *Br. J. Cancer*, **63**, 119–125.

LONG A, PARRICK J AND HODGKISS RJ. (1991). An efficient procedure for the 1-alkylation of 2-nitroimidazoles and the synthesis of a probe for hypoxia in solid tumours. *Synthesis*, 709–713.

LORD EM, HARWELL L AND KOCH CJ. (1993). Detection of hypoxic cells by monoclonal antibody recognizing 2-nitroimidazole adducts. *Cancer Res.*, **53**, 5721–5726.

OVERGAARD J. (1992). Importance of tissue hypoxia in radiotherapy. A meta-analysis of controlled clinical trials. *Radiother. Oncol.*, **24** (suppl.), 64.

RALEIGH JA, FRANKO AJ, KOCH CJ AND BORN JL. (1985). Binding of misonidazole to hypoxic cells in monolayers and spheroid culture: evidence that a side-chain label is bound as efficiently as a ring label. *Br. J. Cancer*, **51**, 229–235.

RALEIGH JA, MILLER GG, FRANKO AJ, KOCH CJ, FUCIARELLI AF AND KELLEY DA. (1987). Fluorescence immunohistochemical detection of hypoxic cells in spheroids and tumours. *Br. J. Cancer*, **56**, 395–400.

RASEY JS, KROHN KA, GRUNBAUM Z, CONROY PJ, BAUER K AND SUTHERLAND RM. (1985). Further characterisation of 4-bromomisonidazole as a potential detector of hypoxic cells. *Radiat. Res.*, **102**, 76–85.

SAPIRSTEIN LA. (1958). Regional blood flow by fractional distribution of indicators. *Am. J. Physiol.*, **193**, 161.

STRATFORD IJ. (1992). Bioreductive drugs in cancer therapy. *Br. J. Radiol.*, suppl. **24**, 128–136.

SZEJTLI J. (1982). Cyclodextrins and their inclusion complexes, In *Carbohydrate Polymers*, **12**, pp. 375–392. Akademiai: Budapest.

URTASUN RC, CHAPMAN JD, RALEIGH JA, FRANKO AJ AND KOCH CJ. (1968). Binding of $^{3}$H-misonidazole to solid human tumours as a measure of tumour hypoxia. *Int. J. Radiat. Oncol. Biol. Phys.*, **12**, 1263–1267.